# A TRPV2 interactome-based signature for prognosis in glioblastoma patients

**DOI:** 10.18632/oncotarget.24843

**Published:** 2018-04-06

**Authors:** Pau Doñate-Macián, Antonio Gómez, Irene R. Dégano, Alex Perálvarez-Marín

**Affiliations:** ^1^ Unitat de Biofísica, Departament de Bioquímica i de Biologia Molecular, Facultat de Medicina, Universitat Autònoma de Barcelona, Bellaterra, Catalonia, Spain; ^2^ Centre for Genomic Regulation (CRG), The Barcelona Institute of Science and Technology, Barcelona, Catalonia, Spain; ^3^ Universitat Pompeu Fabra, (UPF), Barcelona, Catalonia, Spain; ^4^ CIBER Cardiovascular Diseases (CIBERCV), Instituto de Salud Carlos III, Madrid, Spain; ^5^ REGICOR Study Group, Cardiovascular Epidemiology and Genetics Group, IMIM (Hospital Del Mar Medical Research Institute), Barcelona, Catalonia, Spain

**Keywords:** TRPV2, proteomics, glioblastoma multiforme, gene-disease associations, gene signature

## Abstract

Proteomics aids to the discovery and expansion of protein-protein interaction networks, which are key to understand molecular mechanisms in physiology and physiopathology, but also to infer protein function in a guilt-by-association fashion. In this study we use a systematic protein-protein interaction membrane yeast two-hybrid method to expand the interactome of TRPV2, a cation channel related to nervous system development. After validation of the interactome *in silico*, we define a TRPV2-interactome signature combining proteomics with the available physio-pathological data in Disgenet to find interactome-disease associations, highlighting nervous system disorders and neoplasms. The TRPV2-interactome signature against available experimental data is capable of discriminating overall risk in glioblastoma multiforme prognosis, progression, recurrence, and chemotherapy resistance. Beyond the impact on glioblastoma physiopathology, this study shows that combining systematic proteomics with *in silico* methods and available experimental data is key to open new perspectives to define novel biomarkers for diagnosis, prognosis and therapeutics in disease.

## INTRODUCTION

Among transient receptor potential (TRP) channels, the TRP vanilloid 2 (TRPV2) cation channel is a thermal, mechanical, and lipid sensor related to a wide span of physiological roles such as thermogenesis, cardiac structure, neuromuscular development and function, etc. [1–4]. The lack of information on TRPV2 regulation, and about endogenous or pharmacological modulation, has hindered the association of TRPV2 to key human physiopathology processes. To assess TRPV2 function, several attempts have focused on TRPV2 protein-protein interactions (PPI). Some of the interactors for TRPV2 have been inferred through *in silico* methods, using sequence conservation of specific motifs in the TRPV1-4 subgroup [5]. Classical PPI studies have identified TRPV2 partners, such as RGA or NGF-1 [6, 7].

PPI screenings rely in the understanding of a protein-of-interest physiological function using a guilt-by-association approach [8]. These PPI have to be further validated in an appropriate physiological system, becoming the time-limiting step for the discovery of protein-function or protein-disease associations. The molecular identity of the interactors is expected to indicate potential useful mechanisms. In a guilt-by-association fashion the TRPV2-NGF-1 interaction would suggest the role of TRPV2 in neural development, confirming previous results relating TRPV2 to central nervous system (CNS) physiology [7, 9].

*In silico* approaches have taken advantage of gene and protein annotation to computationally refine the number of valid PPI [10, 11]. Availability of experimental data in public databases opens new perspectives for cross-validation of PPI, to assess the robustness of interactomes derived from systematic high-throughput experimental screenings, to associate protein to diseases and to build gene/protein signatures for disease therapeutics, diagnosis and /or prognosis [12].

In this study we propose a guilt-by-association experimental approach to identify TRPV2's PPI aiming to answer physiologically relevant questions for this ubiquitous, but elusive ion channel. Using unsupervised *in silico* approaches we assessed the robustness of the interactome, and then we cross-validated the TRPV2 interactome with disease association databases to define physiopathological implications. Finally, using a patient cohort, we defined a significant TRPV2 interactome-based signature for the prognosis of an important brain disease, such as glioblastoma multiforme (GBM) and we validated the defined signature in two independent cohorts.

## RESULTS

### TRPV2 interactome

**Figure 1:**
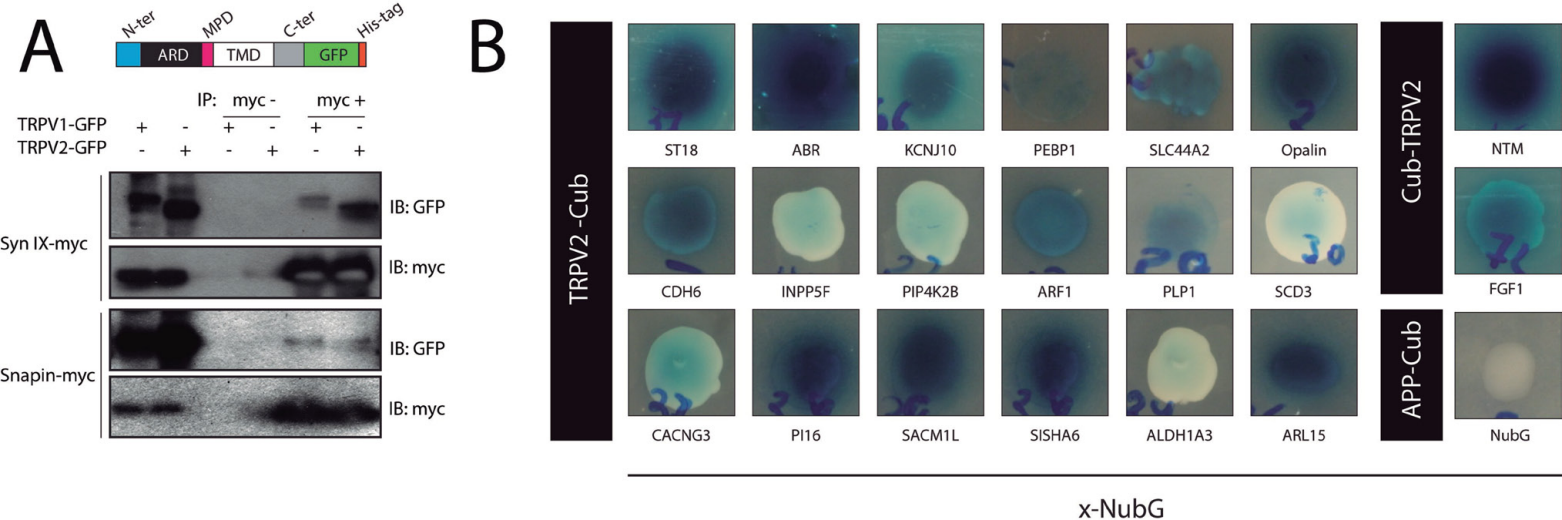
TRPV2 Interactome (**A**) Validation by coimmunoprecipitation of the physical interaction between TRPV2 (GFP-HIS tagged) and synaptotagmin-IX and snapin (Myc tagged) in HEK293 cells. An illustrative cartoon depicts TRPV2 domain organization and the position of the tags. (**B**) TRPV2 interactors discovered in the MYTH assay. The TRPV2 (Cub tagged) and prey (NubG tagged on c terminal, x-NubG) interactions were grown in 10 mM 3AT SD-LEU-TRP-HIS or 5 mM 3AT SD-LEU-TRP-HIS selective plates (Cub-TRPV2 and, TRPV2-Cub respectively) and produced the characteristic blue color from X-Gal metabolism due to expression of B-galactosidase reporter. Colony growth and blue color intensity indicates the strenght of the interaction. As control, we include APP-Cub (amyloid precursor protein), and free NubG as bait and prey plasmids, respectively.

Figure 1A shows the co-immunoprecipitation of TRPV2 with snapin and synaptotagmin-IX in HEK293T cells, using TRPV1 as positive control for the interaction [13]. To define the TRPV2 interactome, we used a split-ubiquitin-based membrane yeast two-hybrid (MYTH) assay [14, 15] to screen a human brain cDNA library, where we identified 20 positive TRPV2 interactors, depicted in Figure 1B and Supplementary Table 1. The positive interactors for human TRPV2 that had highest growth over selective media and strongest blue color intensity in the presence of X-Gal were: ABR, ARL15, NTM, Opalin, SACM1L and ST18 (Figure 1B). The transformants that showed the dimmest blue color intensity were: PIP4K2B, INPP5F, SDC3 and ALDH1A3. These interactors were able to grow under selective conditions, although they did not turn intensely blue in presence of X-Gal (Figure 1B).

### Bioinformatics validation of TRPV2 interactome

**Figure 2:**
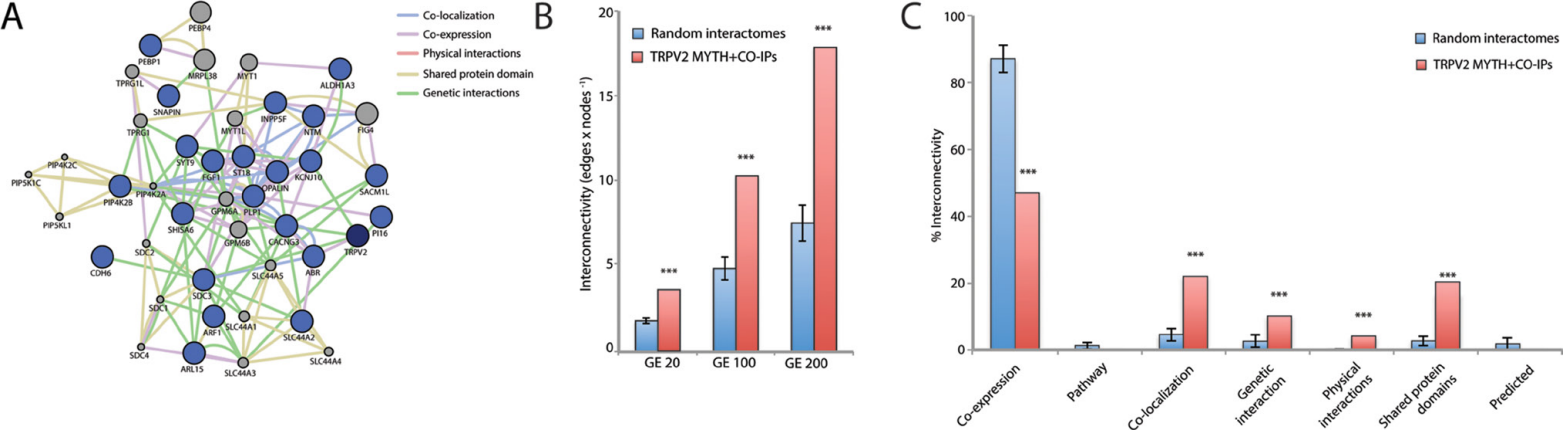
TRPV2 interactome gene enrichment analysis (**A**) Gene enrichment using the 20 closest related genes. The network generated connects TRPV2 MYTH plus CoIP interactors (blue dots) and 20 closest related genes (grey dots) according with the following GO-terms: gene co-expression (purple lines); co-localization (blue lines); genetic interactions (green lines); physical interactions (red lines) and shared domains (brown lines). (**B**) Network interconnectivity measured as the ratio of edges:nodes within the network. TRPV2 interactome from MYTH plus CoIP was compared with randomly generated networks (*n* = 16). The analysis was performed using a gene enrichment of 20, 100 or 200 closest related genes (GE 20, GE100 and GE200 respectively). (**C**) The nature of the network interconnectivity was assessed for TRPV2 interactome and randomly generated networks. The percentage of each type of connection over total connections within the network was represented. ^***^indicates *p*-values < 0.001 between compared groups.

**Figure 3:**
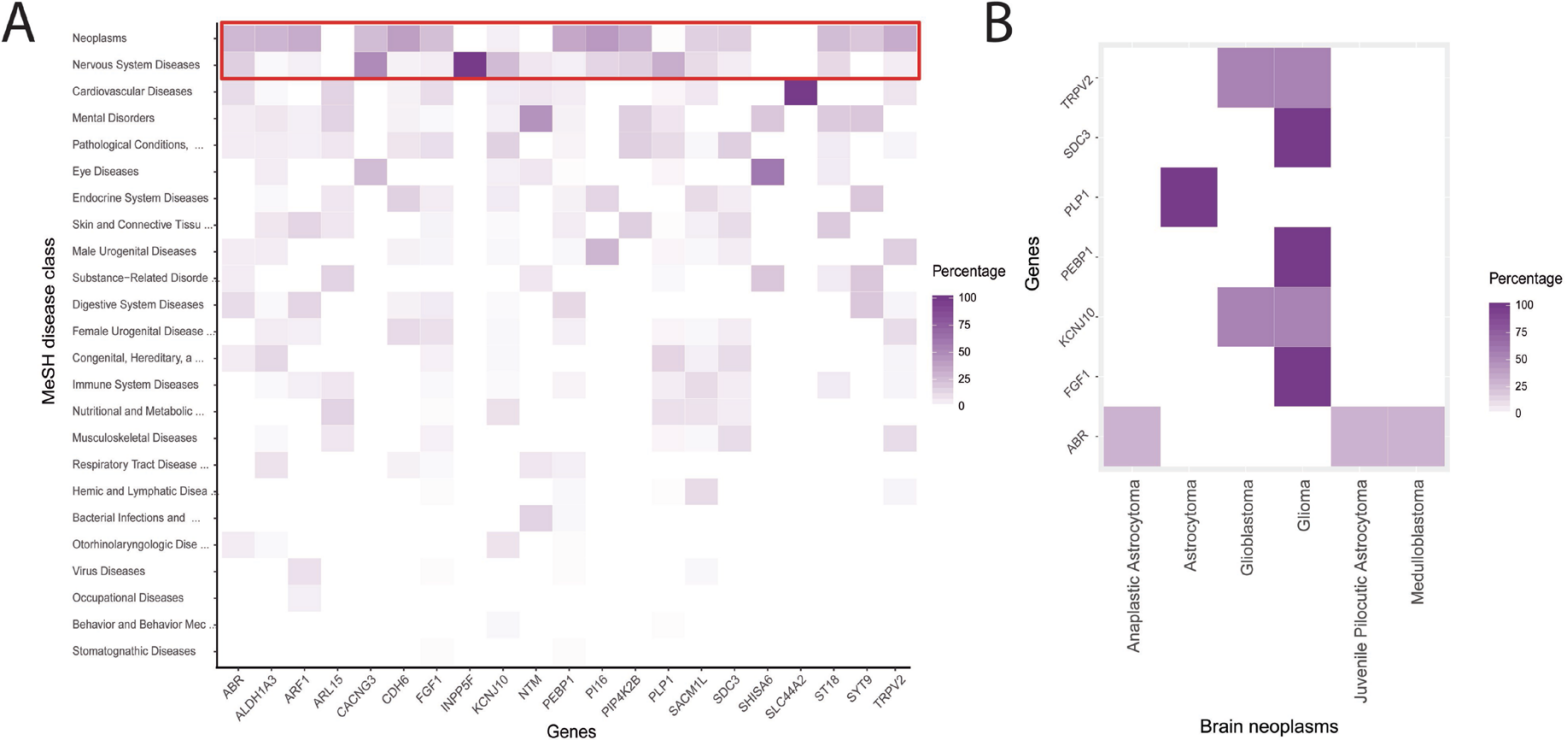
TRPV2 interactome-disease associations (**A**) Single gene-disease associations derived from Disgenet. The red-line frame highlights the top-ranked gene-disease associations. (**B**) Gene-disease associations for CNS/brain neoplasms, according to the 2016 WHO classification for CNS tumors [31] were plotted to show the unique genes previously associated to brain neoplasms.

TRPV2 and its interactors (MYTH hits, snapin, and synaptotagmin-IX) define a putative protein-protein interaction network of 23 proteins (blue nodes in Figure 2A). We expanded the network with the 20-closest genes of the 23 proteins of interest (grey nodes in Figure 2A). The gene-enriched TRPV2 network showed significant higher interconnectivity than 15 randomly generated networks, enriched using 20, 100, or 200 of the network closest-genes (Figure 2B). Regarding the gene ontology terms (GO-terms) used, 4 of them; co-expression, co-localization, genetic and physical interactions, and shared protein-domains terms, are capable of distinguishing the TRPV2 interactome from randomly generated interactomes (Figure 2C and Supplementary Table 2). We browsed in Disgenet for interactome-disease associations [16], rather than unique gene-disease associations, to evaluate the type of tissue-specific disease where our TRPV2-interactome presented a higher association. Neoplasms and nervous system diseases were the top disease classes represented in Disgenet for all the interactome (except for Snapin and Opalin that were not present in any of the gene-disease databases available for Disgenet, Figure 3A). The neoplasms with higher gene-disease associations were metastatic neoplasms and breast-related neoplasms (Supplementary Figure 2). Since the MYTH was performed using a human brain cDNA library, we intersected both neoplasms and CNS diseases to identify the brain neoplasms more associated with our TRPV2 interactome (Figure 3B). The TRPV2 interactome showed a high association with early glia-related neoplasms, especially glioma/glioblastoma, being ABR, FGF1, KCNJ10, PEBP1, PLP1, SDC3, and TRPV2, the genes associated to these brain neoplasms. This subset of proteins defined an initial 7-gene signature for GBM, consisting of genes that have been previously associated to GBM or early stage gliomas, as found in Disgenet (Figure 3B).

### Evaluation of TRPV2 interactome-based signature for prognosis in Glioblastoma patients

**Figure 4:**
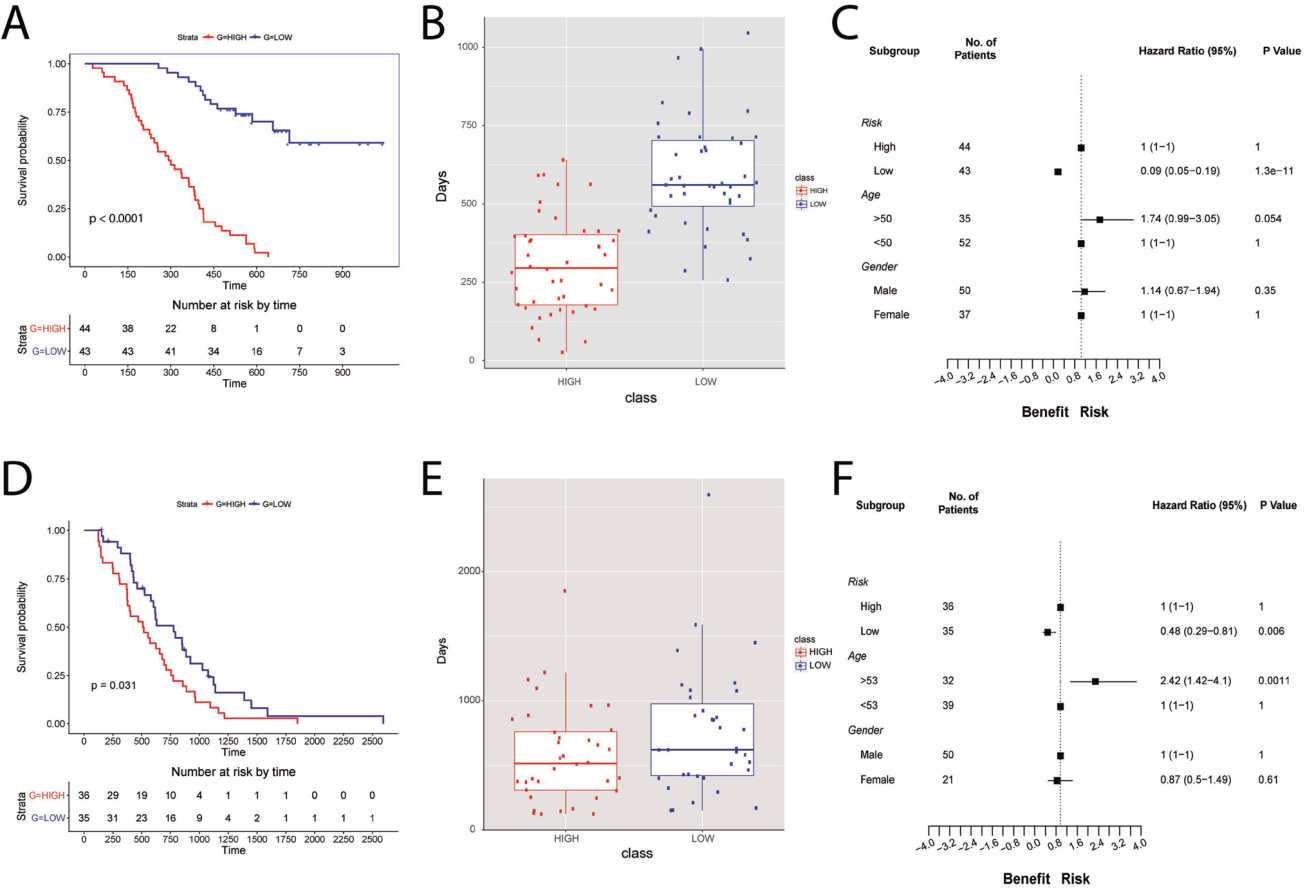
TRPV2 interactome-based glioblastoma signature using the CGGA database for building the signature and the REMBRANDT cohort for the validation of the signature prognostic power (**A**, **D**) Survival plots for low-(blue) and high-risk (red) patients in the CGGA database and REMBRANDT cohort, respectively. (**B**, **E**) Boxplots showing the difference in survival days between high- (red) and low-risk (blue) scores for CGGA database and REMBRANDT cohort, respectively. (**C**, **F**) Forest plots showing hazard ratios with 95% confidence intervals (CI) for CGGA database and REMBRANDT cohort, respectively. The variables used are risk, age, and gender.

The TRPV2 interactome-based signature (all 23 genes) was evaluated in 87 glioblastoma patients from the Chinese Glioma Genome Atlas (CGGA) dataset. Survival of the groups is shown in Figure 4A. Patients with high TRPV2 interactome expression (high-risk scores) had shorter median OS than patients with low expression (low-risk scores) in GBM (multivariate Cox analysis points to risk score as an independent prognostic factor, *p* < 0.001 log-rank test, Figure 4A). Using the median risk score as the cutoff value, the patients were successfully clustered into high- and low-risk groups (Figure 4B). Based on the developed signature, low-risk patients had a lower risk of death compared to high-risk patients in unadjusted analyses (HR = 0.09, 95% CI, 0.05–0.19) (Figure 4C). No significant differences were found by gender while risk of death was borderline higher in older patients (HR = 1.74, 95% CI, 0.99–3.05).

### Validation of the prognostic value of TRPV2 interactome-based signature in additional Glioblastoma datasets

The validation cohort included 71 GBM patients. Consistent with the results from the CGGA cohort, the signature successfully discriminated patients with poor prognosis (high-risk) from those with a better prognosis (low-risk In the REMBRANDT dataset, Figure 4D, 4E). Multivariate Cox analysis indicated that the risk score was an independent prognostic factor of OS (*p* < 0.05 log-rank test) (Figure 4D). The trend to a higher risk of death observed in CGGA older patients was also found significant in REMBRANDT (HR = 2.42, 95% CI, 1.42–4.1, Figure 4F). GBM patients, as other type of neoplasm, show altered DNA repair pathways leading to chemotherapy resistance. Temozolomide sensitivity has been related to overexpression of O^6^-methylguanine methyltransferase (MGMT) and/or lack of DNA repair [17]. Using the REMBRANDT cohort, we could study the relationship between MGMT, base excision repair (BER) genes, and TRPV2, to infer whether our TRPV2-interactome signature could be of use to assess chemotherapy resistance in GBM patients. The OS in this subset indicates that the risk score is an independent prognostic factor (*p* = 0.00011 log-rank test, Supplementary Figure 3A), and the low risk group shows overexpression of MGMT and TRPV2 and underexpression of BER genes (Supplementary Figure 3B).

**Figure 5:**
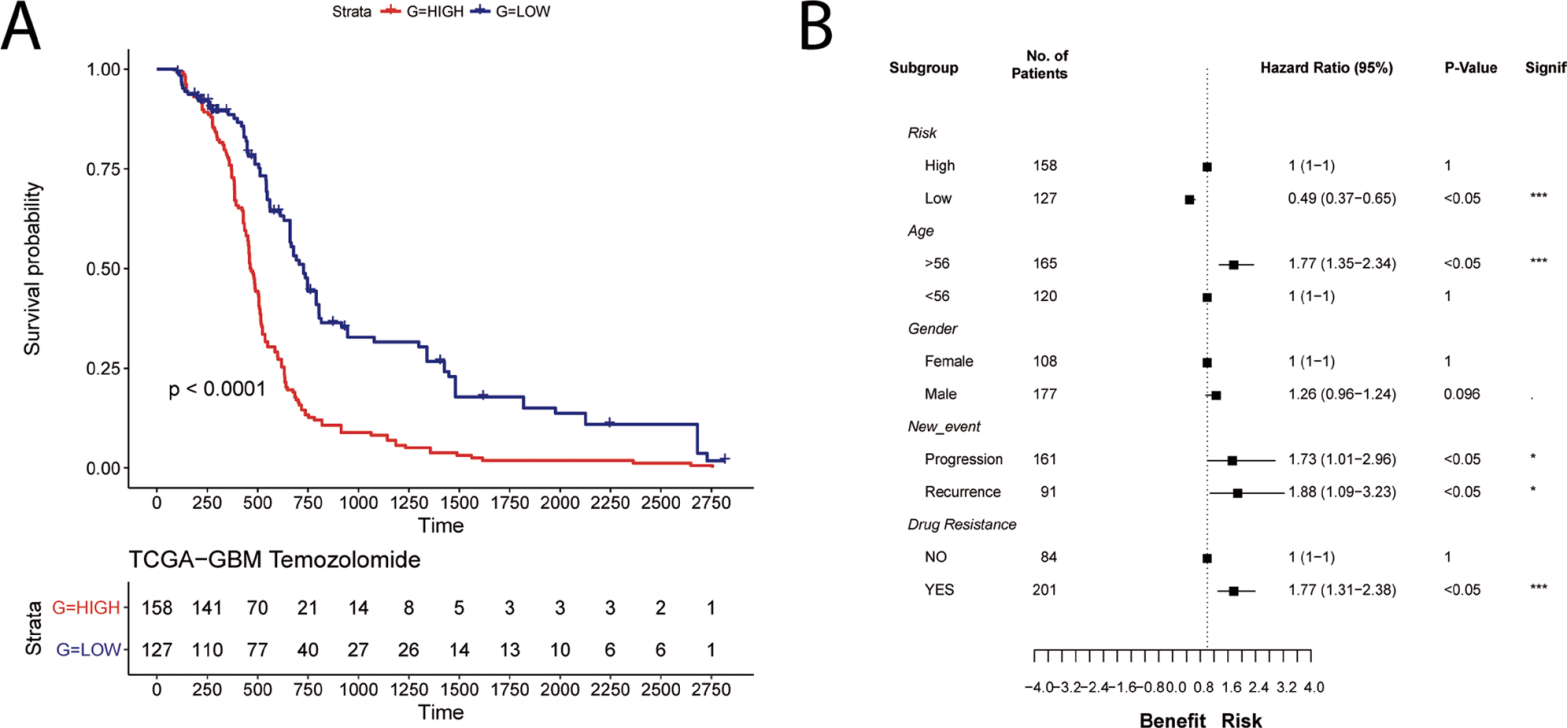
Validation of the prognostic power of the TRPV2 interactome-based glioblastoma signature in the TCGA cohort with chemotherapy and tumor progression information (**A**) Survival plot in low-(blue) and high-risk (red) patients classified by the TRPV2 interactome. (**B**) Forest plots showing hazard ratios with 95% confidence intervals (CI) for the variables risk, age, gender, new event, and drug resistance.

We further tested the robustness of the TRPV2-interactome signature using another independent cohort with drug resistance (Temozolomide), progression and recurrence information, the Cancer Genome Atlas (TCGA) GBM data of 285 patients. Kaplan-Meier plots showed significant differences in OS between the low and high-risk groups (HR = 0.49, 95% CI, 0.37–0.65; Figure 5A). Moreover, significant differences are shown regarding age, and Temozolomide resistance, but also for GBM progression and recurrence (Figure 5B).

### Functional annotation of the interactome signature

In order to find out the functional basis of the notable difference in prognosis, we also performed differential expression analysis on high- and low-risk patients in the three datasets. Those genes with false discovery rate (FDR) < 0.05 were considered to be differentially expressed between the two groups (Supplementary Table 3). The overlapped genes (Supplementary Table 4) were chosen for further analysis. Gene ontology (GO) enrichment analysis revealed that the associated genes, among those highly expressed in the high-risk group, were primarily associated with the nervous system development, neuron differentiation, regulation of cell morphogenesis, axon development, cell morphogenesis involved in neuron differentiation, and synaptic transmission and signaling (Supplementary Figure 4), agreeing with the bioinformatics validation of the interactome (see above).

## DISCUSSION

In this study, we used a guilt-by-association discovery approach to unravel the role of TRPV2 and its PPI in CNS pathophysiology. The putative TRPV2 interactome consists of TRPV2 + 22 proteins derived from canonical immunoprecipitation studies and a systematic PPI screening among which ABR, ARL15, NTM, Opalin, SACM1L and ST18, seem to have the strongest interaction with TRPV2. The defined TRPV2 interactome showed higher interconnectivity than randomly generated networks and 4 GO terms allowed its differentiation from random interactomes. The most associated diseases with the TRPV2 interactome were neoplasms and diseases from the nervous system, and ABR, FGF1, KNJ10, PEBP1, PLP1, SOC3, and TRPV2 were found to be associated with brain neoplasms. Expression of the TRPV2 interactome was significantly associated with less survival of glioblastoma patients in a derivation and 2 validation cohorts. In addition, high expression of the TRPV2 interactome showed a higher risk for older individuals and was associated with progression, recurrence, and drug resistance.

Our study identified a set of 22 proteins as potential interactors of TRPV2. The proteins present in our interactome include transport proteins (TRPV2, KCNJ10, CACNG3, SLC44A2), catalytic proteins (ALDH1A3, INPP5F, PIP4K2B), trafficking proteins (ARF1, ARL15, ABR, SYT-9, SNAPIN), signaling molecules (FGF1, ST18, SHISA6, CDH6, SDC3), myelin components (PLP1, Opalin, NTM), lipid metabolism proteins (SACM1L, PEBP1, INPP5F, PIP4K2B), etc. From the identified proteins ABR, ARL15, NTM, Opalin, SACM1L and ST18, appear to have the strongest interaction with TRPV2. Using a gene-enrichment FDR approach, we showed that TRPV2 interactome interconnectivity was significantly larger than randomly generated interactomes. The higher interconnectivity relates to the robustness of the experimental set of interactors for TRPV2, indicating low or none contribution of false positive interactors to the TRPV2 network. Thus, if the interactome is robust as the identified TRPV2 interactome, it should behave as a functional unit in physiological or physiopathological conditions. The gene-enrichment analyses highlighted specific GO-terms, such as protein co-localization, protein shared domains, and protein physical interactions. These GO-terms point to molecular mechanisms, rather than single gene-functions in line with the different functions of the interactome proteins. This approach does not validate the physical interactions within TRPV2 interactome *per se*, but argues for the robustness of the interactome and indicates a high degree of relationship between the proteins and/or their biochemical function, similarly to the scheme followed by *de novo* and *in silico* PPI predictors [10, 18]. Our *in silico* validation does not prevent the experimental validation, but serves as a tool to rank the interactors by specific GO-terms of interest and highlight the robustness of the interactions.

Assuming the TRPV2 interactome as a whole, instead of a sum of proteins, we have used it as input seed to obtain interactome-disease associations in Disgenet. To the best of our knowledge, this is the first time that this approach has been used. This analysis identified neoplasms and nervous system diseases as the most associated diseases with the TRPV2 interactome, and highlighted a set of genes previously associated to brain neoplasms, such as: ABR, FGF1, KCNJ10, PEBP1, PLP1, SDC3 and TRPV2. TRPV2 is overexpressed in GBM [19] and it has been shown that its activation by cannabidiol sensitizes GBM cells to chemotherapeutics and also inhibits proliferation of Glioblastoma stem-cell like cells [20, 21].

GBM is the most common primary brain neoplasm and one of the worst in terms of treatment and prognosis, with an incidence of 3 cases per 100,000 people and a median survival of 12 months [22, 23]. Many efforts are directed towards the understanding of the malignancy of glioblastoma to find therapeutic and diagnostic tools to improve its prognosis. Gene-based signatures, opposite to single gene expression, have been widely used to understand neoplasms [12, 24]. A top-down supervised study based on the expression levels of ion channels in GBM identified a three-gene signature (KCNN4, KCNB1 and KCNJ10) significantly associated with OS of GBM [25]. Our main result is a proteomics interactome-based signature for GBM prognosis derived in a GBM patient cohort and validated in 2 independent cohorts. The interactome-based signature significantly discriminates among high- and low-risk GBM in terms of OS time, but also in terms of GBM progression, recurrence, and Temozolomide resistance. The results of the validation cohorts demonstrate the robustness of the signature to identify patients at high-risk of short survival and disease recurrence. Our results also suggest a benefit of adjuvant therapy for the TRPV2-interactome signature. The identified TRPV2-interactome-based signature represents a new set of potential biomarkers not only for the study of GBM, but for prognosis and therapeutics of one of the most malignant neoplasms.

In conclusion, we have identified a set of TRPV2 interactors using biochemical and computational analyses. This TRPV2 interactome is robust and it is particularly associated with neoplasms and diseases of the nervous system. Seven of these interactors have been associated with brain neoplasms in previous studies. Analyses in 3 different cohorts have shown that the identified TRPV2 signature is able to discriminate between glioblastoma patients at low- and high-risk in terms of survival. The TRPV2 signature is also associated with glioblastoma patient age, disease progression, recurrence, and drug resistance. Our results provide a protein signature that could be useful for GBM prognosis and treatment. Due to the potential implications of our work larger studies are needed to confirm the observed associations.

## MATERIALS AND METHODS

### DNA plasmids and cell culture and transfection

cDNA sequences for rat TRPV1 and TRPV2 cloned into a pCDNA3 vector and Myc-his tagged snapin and synaptotagmin-IX cloned in a pcDNA3 vector were transiently transfected in cultured HEK 293T cells as described in Doñate-Macián *et al.* [26]. HEK293 cells overexpressing the transfected constructs were harvested 48 hours after transfection and proteins solubilized in lysis buffer (50 mM Tris-HCL pH 7.4, 150 mM NaCl, 2 mM EDTA, 1% Triton-X100, 5% glycerol, 1 mM benzamidine and EDTA-free protease inhibition cocktail, ROCHE).

PBT3-STE and pBT3-N yeast expression vectors were used in the MYTH assay to express human TRPV2 as bait (tagged with the C-terminus of split-ubiquitin at the C- and N-terminus, respectively) and a cDNA library from human brain (Dualsystems Biotech P12227, tagged with the mutated N-terminus of split-ubiquitin NubG). pTSU-APP (amyloid precursor protein), and pPR3-N were used as control bait and control prey plasmids, respectively. Human TRPV2 full length, tagged with an eGFP at the C-terminus was cloned into pBT3-N and pBT3-STE within SfiI sites to monitor cellular expression.

### Immunoprecipitation

500 μg of total protein from the cell extracts were incubated overnight at 4° C with anti-c-Myc antibody (BD Pharmingen, 551101). 50 μL of Sepharose-G beads were added and incubated for 2 hours at 4° C. Immunoprecipitated complexes were denatured with SDS-PAGE sample buffer (90° C for 5 min), separated by SDS-PAGE and analyzed by immunoblotting as previously described [26] using as primary antibodies anti-GFP from Developmental Studies Hybridoma Bank (DHSB GFP-G1) 1:1000 dilution and c-Myc (BD Pharmingen, 551101) 1:1000 dilution. Secondary antibody (Santa Cruz, anti-mouse sc-2031) 1:3000 dilution was incubated 1 hour at room temperature. Detection was carried out with Illumina Crescendo reagent (Millipore).

### Membrane yeast two-hybrid assay

MYTH assay as described by Snider *et al.* [27] was used to identify potential interactors for TRPV2. MYTH assay was performed in the reporter strain NMY51 [*MATa his3delta200 trp1-901 leu2-3,112 ade2 LYS2::(lexAop)4-HIS3 ura3::(lexAop)8-lacZ (lexAop)8-ADE2 GAL4)*] (Dualsystems Biotech, P01401-P01429). Western blot against LexA tag (SC-7544HRP, Santa Cruz Biotech) was used to confirm the correct expression of the generated baits. NMY51 yeast cells expressing GFP tagged baits were visualized using a Leica TCS SP5 confocal microscope (Supplementary Figure 1). Transformation efficiency of the human brain cDNA library into NMY51 cells carrying the bait constructs was assessed by counting the number of colonies growing on non-selective plates (SD-LEU-TRP). Transformation efficiency was higher than 2 × 10^6^ colony forming units. Positive transformations were plated into SD agar selective plates as follows: LexA-VP16-Cub-TRPV2 (subsequently called Cub-TRPV2) was plated in 10 mM 3AT SD-LEU-TRP-HIS and TRPV2-Cub-LexA-VP16 (subsequently called TRPV2-Cub) in 5 mM 3AT SD-LEU-TRP-HIS. After incubation at 30° C for 3–5 days, transformants were transferred to same stringency selective plates containing 5-bromo-4-chloro-3-indolyl-β,d-galactopyranoside (X-Gal) to assess B-galactosidase reporter gene expression. Prey plasmids DNA were isolated and transformed into DH5α *E. coli* competent cells to be sequenced. Isolated prey plasmids in frame with NubG carrying a putative interaction partner were transformed again into NMY51 strain containing the original bait and plated into selective plates with X-gal to confirm the interaction. Refer to Supplementary Figure 1 for further experimental details.

### Interactome robustness and interconnectivity: gene enrichment and network analysis

Genes determined by the MYTH assay including TRPV2 (21 hits) and the 2 Co-IP hits were used to construct a network in Cytoscape [28] with the Genemania algorithm [29]. The network was constructed using as template the Genemania Human genome and adding the 20, 100 or 200 closest related genes (GE20, GE100 and GE200 respectively) based on the following GO terms: colocalization, co-expression, physical interactions and domain conservation. An equal number of random genes (23) from human genome were used to construct networks using the same Genemania GO-terms. A total of 16 random networks were generated by this approach. Nodes and edges within each network were quantified and the ratio edges:nodes was used as a measure of network connectivity.

### Disgenet analysis

The disgenet2r R package [30] was used to obtain the genes associated to neoplasms. A list of genes was created with the 23 interactors identified. All genes except for Opalin and Snapin were identified in the Disgenet database [16]. Gene-disease associations were obtained with the disgenetGene function and extracted to a data frame. Gene-disease associations for neoplasms and CNS/brain neoplasms, according to the 2016 WHO classification for CNS tumors [31] were plotted to show the genes associated to each disease.

### Data integration and signature development

All clinical and gene expression data were collected previously and were available from public databases. Three databases were used in the analysis: CGGA (http://www.cgga.org.cn), TCGA (http://cancergenome.nih.gov), and Repository for Molecular Brain Neoplasia Data REMBRANDT (E-GEOD-68848, http://www.ebi.ac.uk/arrayexpress/). Specifically, in this study, we used data from three platforms: Affymetrix HG-U133Plus2 (REMBRANDT) Agilent Whole Human Genome Oligo Microarray in a 4^*^44 (CGGA), and RNAseq (TCGA). Raw data (CEL files) for REMBRANDT data set were processed with the statistical algorithm Robust Multiarray Average (gcRMA) with the affy package of R/Bioconductor [32, 33]. Microarray analysis of CGGA glioma samples was performed with the Agilent Whole Human Genome Array, according to the manufacturer's instructions. Both in REMBRANDT and CGGA datasets, average values of replicate spots for each gene were background subtracted, normalized, log2-transformed, for further analysis. TCGA level 3 gene expression levels (RNAseq) were obtained from the TCGA Data Portal in March 2017, in order to compare to microarray data, this data was normalized and log2-transformed as well.

87 patients with microarray and clinical data available from CGGA were used as the training dataset, and the TCGA and REMBRANDT patients with all required information (285 and 71, respectively) were used as the validation datasets.

The 23 proteins were applied as a signature to develop a risk score (RS) formula in glioblastoma patients from the CGGA dataset:

$$RS=\sum_{i=1}^{n}\left(\beta_{l}\cdot \exp r\_level_{i}\right)$$

Where β corresponds to the regression coefficient from the univariate Cox analysis to weigh the respective gene expression level (expr_level). Patients were stratified into high- and low-risk by using the 50th percentile risk score as the cut-off.

Genes which were associated with grade progression and significant prognostic value (*p*-value < 0.05) were used to developed a linear combination of the gene expression level (expr_level) weighted by the regression coefficient derived from the univariate Cox regression analysis (β). For the signature validation, the β obtained in the derivation dataset were used and patients were also stratified into high- and low-risk as described above.

### Statistical analysis

All statistical analysis were performed using R Statistical Software (version 3.2.1; Foundation for Statistical Computing, Vienna, Austria) [34].

Network interconnectivity from randomly obtained networks was compared to the TRPV2 generated network and significant differences were evaluated using *T*-test. *P*-values of GO-terms were calculated with the hypergeometric test and corrected with the Benjamini & Hochberg FDR algorithm.

Analysis of differentially expressed genes was performed using the limma package in R [35]. Briefly, linear models were used to assess gene differential expression in the TRPV2-interactome gene set for multifactor data available in the TCGA, CGGA, and REMBRANDT databases.

Uni- and multivariate survival analyses were carried out with the R package Survival [36, 37] by performing Kaplan–Meier analyses with the log-rank test after the proportional hazards assumption was verified. Kaplan-Meier curves were calculated to obtain survival estimates and the log-rank test to examine the differences between groups. OS was calculated as the interval from the day of first surgery to death or the end of follow-up. Multivariate survival analyses (Cox proportional hazard model) were used to take into account the joint effect of different covariates in OS. The included covariates were drug resistance, tumor progression and recurrence, gender, and age. Forest plots showing the hazard ratios (including 95% confidence intervals) for all cohorts were produced using R [34].

## SUPPLEMENTARY MATERIALS FIGURES AND TABLES











## Abbreviations

CGGA: Chinese Glioma Genome Atlas
FDR: false discovery rate
GBM: glioblastoma multiforme
GO-term: gene ontology term
MYTH: membrane yeast two-hybrid
OS: overall survival
PPI: protein-protein interactions
TCGA: the Cancer Genome Atlas
TRP: Transient receptor potential
TRPV2: Transient receptor potential vanilloid 2
