# Supplementary material for: A TRPV2 interactome-based signature for prognosis in glioblastoma patients
Source: Oncotarget. 2018 Apr 6;9(26):18400–9. doi: 10.18632/oncotarget.24843 (PMC5915080; doi:10.18632/oncotarget.24843)
Supplement: Supplementary file 1 [file oncotarget-09-18400-s001.pdf]

A TRPV2 interactome-based signature for prognosis in glioblastoma patients

SUPPLEMENTARY MATERIALS

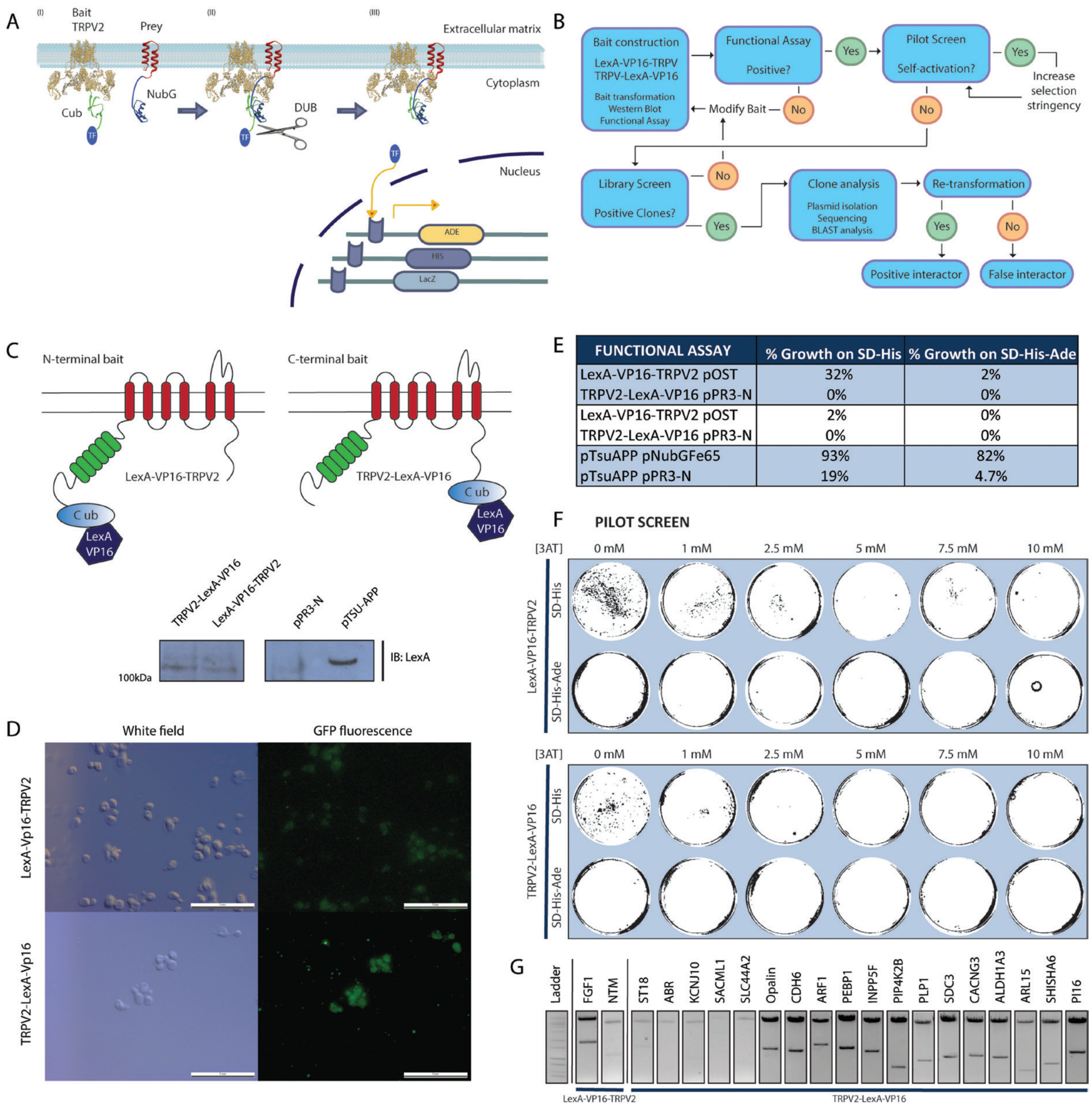

FUNCTIONAL ASSAY	% Growth on SD-His	% Growth on SD-His-Ade
LexA-VP16-TRPV2 pOST	32%	2%
TRPV2-LexA-VP16 pPR3-N	0%	0%
LexA-VP16-TRPV2 pOST	2%	0%
TRPV2-LexA-VP16 pPR3-N	0%	0%
pTsuAPP pNubGFe65	93%	82%
pTsuAPP pPR3-N	19%	4.7%

Supplementary Figure 1: Membrane yeast two hybrid assay (MYTH) for TRPV2. (**A**) MYTH workflow outline: I) TRPV2 bait tagged with the C-terminus of ubiquitin (Cub) and the transcription factor (TF/ VP16LexA) is cotransformed into NMY51 yeast strain with a library of prey cDNAs tagged with N-terminus of ubiquitin (NubG). II) Upon bait and prey interaction the fragments of ubiquitin are refolded and the degradation ubiquitin complex (DUB) releases the TF. III) TF travels to the nucleus and starts the expression of reporter genes that confer the strain resistance to growth on medium lacking adenin (ADE) and histidin (HIS) and the ability to metabolize X-Gal producing a characteristic blue color due to the expression of LacZ gene. (**B**) MYTH overview. The screen is divided into three major

parts: I) Bait construction, expression and verification, II) Library transformation and selection of positive clones, III) Confirmation of interactors and sequence analysis. (**C**) Scheme of the TRPV2 baits tagged with Cub-VP16-LexA on the N and C terminus and WB against LexA confirming the correct expression of both constructs in yeast. (**D**) Fluorescence microscopy showing the correct expression of TRPV2 tagged with Cub-VP16-LexA on the N and C terminus. (**E**) Functional assay to assess the reporter gene activation. Baits are transformed with pOST, a prey that interacts ubiquitously with any bait, and pPR3-N, a prey thatis not able to interact with any bait. pTSUAPP and pNubGfe65, a bait-prey already described interactors, are used as control of the assay. The growth is measured as a percentage of the number colonies that grow on plates lacking HIS and ADE divided by the number of colonies found on plates that do not lack HIS and ADE. (**F**) Pilot screen to set assay stringency. Baits are transformed with pPR3-N, a prey that is notable to interact with any bait and plated on plates lacking HIS or lacking ADE and HIS. The stringency was increased by adding 3-aminotriazole (3-AT). 3-AT is a competitive inhibitor of the HIS3 gene product. The number of colonies is evaluated to choose the proper selective media. (**G**) Agarose gel of purified prey plasmids isolated from the library screen and digested with SfiI to monitor the size of the cDNA.

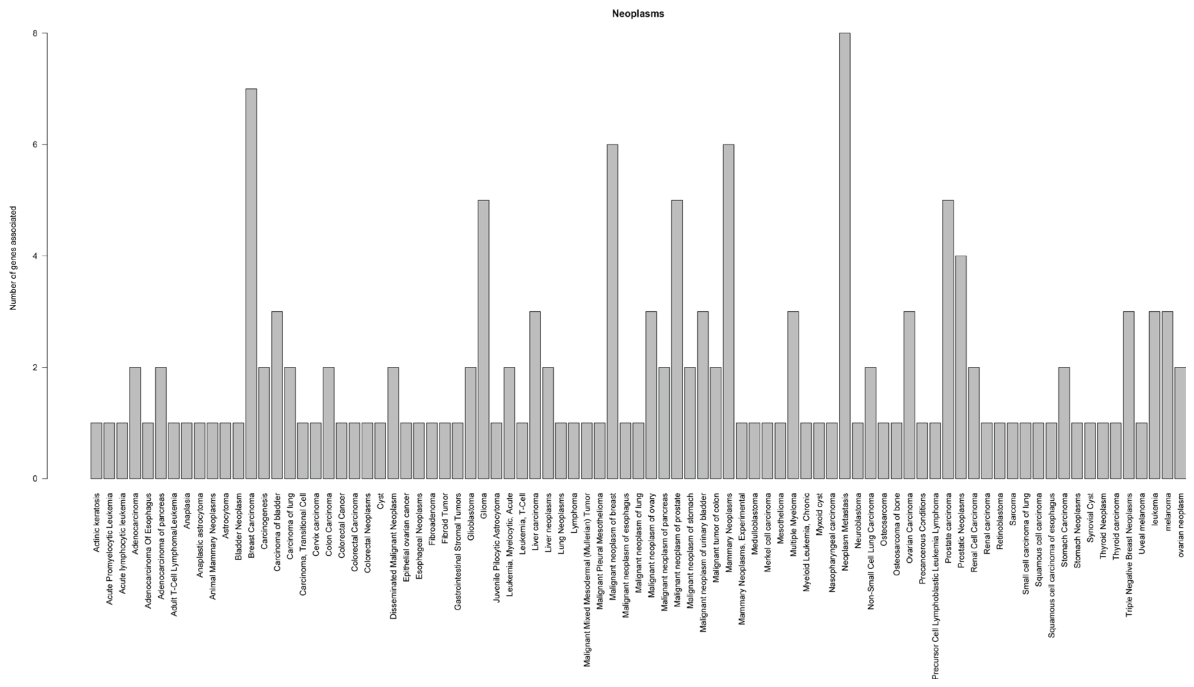

Supplementary Figure 2: Neoplasms with higher gene-disease associations in Disgenet.

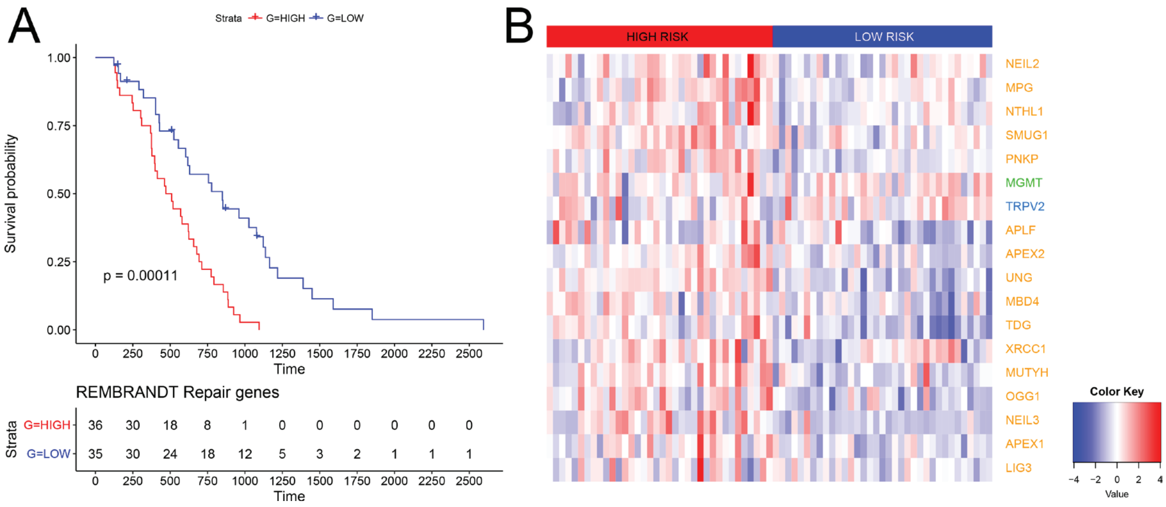

Supplementary Figure 3: Evaluation of the prognostic power of the association between TRPV2 (blue), MGMT (green) and Base Excision repair genes (BER, in orange) expression in the REMBRANDT cohort. (**A**) Survival plot. (**B**) Heatmap showing the differences in expression between high and low risk. Methodology: We selected all the genes in Base Excision repair genes Pathway [1] including MGMT and TRPV2. The analysis of differentially expressed genes was performed using the limma package of R. [1] https://www.mdanderson.org/documents/Labs/Wood-Laboratory/human-dna-repair-genes.html#BER.

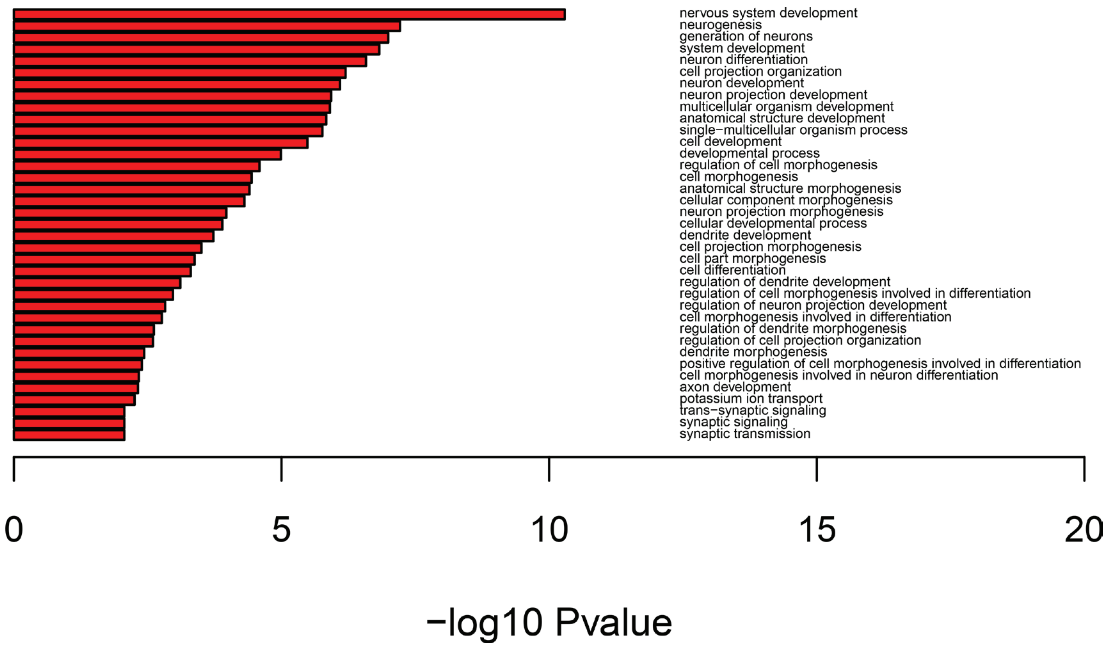

Supplementary Figure 4: GO enrichment analysis for gene/high-risk group association. GO-terms in the high-risk group ranked by log *p*-value.

Supplementary Table 1: MYTH screen data. See Supplementary_Table_1

Supplementary Table 2: Bioinformatics validation of TRPV2 interactome. Genemania output. See Supplementary_Table_2

Supplementary Table 3: Functional annotation of the interactome signature. FDR-refined differentially overexpressed genes. See Supplementary_Table_3

Supplementary Table 4: Functional annotation of the interactome signature. GO-term enrichment of differentially overexpressed genes in the high-risk group. See Supplementary_Table_4
